# Training Schools for Nurses and Their Results

**Published:** 1883-12

**Authors:** 


### Training Schools for Nurses and Their Results.

Next in importance to the subject of the higher education of the medical profession, is that of a thoroughly trained body of nurses. This subject has been receiving special attention from the profession for the last decade, and in many of the large centres of population, schools for the training of women for the proper care of the sick have been organized and are in successful operation. Buffalo is not behind in this excellent work. The Training School for Nurses connected with the Buffalo General Hospital has been in successful operation for several years, carrying the blessings of skilled nursing to the profession at large and to the bed-side of the sick and dying. It is truly a philanthropic work, and is finding an appreciative greeting in the medical profession, who have had to labor against prejudice from within and ignorance without the sick chamber in their work to alleviate human suffering. We have had personal knowledge of the qualifications of the nurses sent forth from the Buffalo Training School, and can testify to the excellency of the care they bestow upon the sick, and the wise judgment they exercise in the absence of the medical attendant. We may truly say their presence is the source of relief from anxiety to the physician, and their employment is also a source of both safety and economy to the patient. We learn that the Training School is attended by a larger number of women now than ever before in its history, and we regard this fact as an auspicious one, indicating an appreciation of its good work on the part of the profession and the public.

